# Can We Increase Psychological Well-Being? The Effects of Interventions on Psychological Well-Being: A Meta-Analysis of Randomized Controlled Trials

**DOI:** 10.1371/journal.pone.0158092

**Published:** 2016-06-21

**Authors:** Laura A. Weiss, Gerben J. Westerhof, Ernst T. Bohlmeijer

**Affiliations:** Centre for eHealth and Well-being Research, Department of Psychology, Health and Technology, University of Twente, Enschede, The Netherlands; University of Pennsylvania, UNITED STATES

## Abstract

**Background:**

There is a rapidly growing interest in psychological well-being (PWB) as outcome of interventions. Ryff developed theory-based indicators of PWB that are consistent with a eudaimonic perspective of happiness. Numerous interventions have been developed with the aim to increase PWB. However, the effects on PWB measured as coherent outcome have not been examined across studies yet. This meta-analysis of randomized controlled trials of behavioral interventions aims to answer the question whether it is possible to enhance PWB.

**Methods:**

A systematic literature search was performed in PsycINFO, Cochrane and Web of Science. To be included, studies had to be randomized controlled trials of behavioral interventions with psychological well-being as primary or secondary outcome measure, measured with either Ryff’s Psychological Well-Being Scales or the Mental Health Continuum—Short Form. The meta-analysis was performed using a random effects model. From the 2,298 articles found, 27 met the inclusion criteria. The included studies involved 3,579 participants.

**Results:**

We found a moderate effect (Cohen’s d = 0.44; z = 5.62; p < .001). Heterogeneity between the studies was large (Q (26) = 134.12; p < .001; I^2^ = 80.62). At follow-up after two to ten months, a small but still significant effect size of 0.22 was found. There was no clear indication of publication bias. Interventions were more effective in clinical groups and when they were delivered individually. Effects were larger in studies of lower quality.

**Conclusions:**

It appears to be possible to improve PWB with behavioral interventions. The results are promising for the further development and implementation of interventions to promote PWB. Delivering interventions face-to-face seems to be the most promising option. We recommend to keep including clinical groups in the research of psychological well-being. Heterogeneity is a limitation of the study and there is need for more high-quality studies.

## Introduction

In the last years, the focus in mental healthcare and prevention has shifted from solely treating or preventing mental health complaints to enhancing positive aspects of mental health. A new goal in mental healthcare is the promotion of well-being [1–4]. However, there are currently many definitions of well-being [5]., with the two main concepts being subjective and psychological well-being.

Subjective well-being builds on a hedonic framework in which striving for positive experiences is central. It is usually measured as satisfaction with life in combination with a balance between positive and negative emotions [6]. The standards that people use to judge their subjective well-being were not theorized in this framework. By contrast, Carol Ryff introduced the concept psychological well-being with the intention to develop theory-based indicators of positive human functioning that were consistent with a eudaimonic perspective of happiness [7]. Another well-researched theory in the eudaimonic tradition is the self-determination theory that states that the fulfillment of basic psychological needs is essential to well-being and growth [8].

The variety of concepts and measures makes it difficult to compare studies [9]. It is therefore important to be precise in one’s definition of well-being. This paper focuses on the concept of psychological well-being according to Ryff’s definition [10]. Earlier meta-analyses have already examined subjective well-being [11, 12]. The latest meta-analysis has also included psychological well-being, but measured it in a very broad way with many different instruments [12]. We will conduct the first meta-analysis that exclusively examines psychological well-being as defined by Ryff.

Based on an extensive review of the literature of clinical, humanistic and life-span developmental psychology, as well as existential and utilitarian philosophy, Ryff [10] defined psychological well-being as a process of self-realization, consisting of six dimensions: autonomy, environmental mastery, personal growth, positive relations with others, purpose in life and self-acceptance. There is some discussion on the six-factor structure [13] and whether psychological and subjective well-being are two separate but related dimensions or one overarching construct [14].

Recently Ryff [15], reviewed over 350 empirical studies on psychological well-being that have been conducted in the past decades. Longitudinal studies show that high levels of psychological well-being are a protective factor against mental illnesses and psychopathology [16–18] and that it is also related to biological markers of physical health, reduced risk for various diseases such as Alzheimer’s disease, and a longer life-duration [15]. This growing evidence of positive outcomes of psychological well-being makes it worthwhile to study whether we can improve it.

However, as existing studies show that psychological well-being is rather stable across time [19], an important question is whether it can indeed be promoted in interventions. Answering this question will provide more insight into the state or trait discussion whether characteristics of psychological well-being are more trait-like or state-like [20].

In recent years, there has been a rapid increase of studies on behavioral interventions that included psychological well-being as an outcome measure (e.g. [21, 22]). A central aim of interventions such as well-being therapy [23, 24], acceptance and commitment therapy [25], life-review therapy [26], and positive psychological interventions [27] is to enhance positive psychological functioning. Meta-analyses have shown that these interventions are successful in enhancing certain aspects of psychological well-being [11, 12, 28], but as mentioned, they measured psychological well-being with many different measurement instruments that do not all fit the definition of Ryff. To which extent interventions have an impact on psychological well-being as a coherent construct of positive psychological functioning is unclear. Also, only positive psychological interventions were included, thereby neglecting the increasing number of interventions that addressed psychological well-being in other disciplines.

Hence, we will take the next step in reviewing the evidence on psychological well-being by conducting a meta-analysis on the effects of different behavioral interventions on psychological well-being as a coherent construct across randomized controlled trials. We want to examine whether well-being can be changed as a function of behavioral interventions.

## Methods

### Eligibility criteria

#### Study eligibility criteria

The research question and inclusion criteria were established before the meta-analysis was conducted. Psychological well-being had to be used as primary or secondary outcome measure. To examine it as coherent construct, it had to be measured either with Ryff’s Psychological Well-Being Scales (PWBS) [10] with all six dimensions of psychological well-being as study endpoints, or with the subscale ‘Psychological Well-Being’ of the Mental Health Continuum—Short Form (MHC-SF) [29, 30]. The MHC-SF also assesses psychological well-being with the six dimensions of Ryff’s model. If the MHC-SF was used, the data of the subscale psychological well-being had to be available. Research on the MHC-SF in different cultures has provided support for its psychometric properties and its three dimensional factor structure [31, 32]. The reliability and validity of the PWBS has been established in different versions and across various cultures (e.g. [33, 34]). Yet it has to be noted that the a priori six-factor structure is debated [13]. This problem appears to be exacerbated by the existence of multiple forms of the test, ranging from 18 to 120 items. There is also discussion whether the PWBS is able to discriminate between higher levels of well-being [35].

Only randomized controlled trials (RCTs) of behavioral interventions were included, excluding pharmacological interventions. We focused on all study populations, including both healthy and clinical populations of any age. Waiting list, no treatment, care-as-usual, placebo, or alternative treatment groups were included as comparators.

#### Report eligibility criteria

To be included, an article had to be published in English-language peer-reviewed journals, excluding books, dissertations and conference proceedings. No publication date restriction was imposed. Data necessary to calculate the effect size had to be available in the article or upon request.

### Search strategy and selection of studies

#### Information sources

A systematic literature search was performed in the databases of the Cochrane Library, PsycINFO, and Web of Science. The last search was run on 13 April 2015. The first and second author developed the search with the help of an information specialist. The first author (LAW) and a trained student assistant (PDW) conducted the search. We screened the reference lists of included studies and of the meta-analyses of Sin and Lyubomirsky (11), Bolier et al. [12] and the review of Ryff [15] for additional potentially eligible studies. Finally, we invited four experts in the field to suggest additional studies that might meet the inclusion criteria.

#### Search

Search terms were Ryff* or "mental health continuum" or "psychological well-being" or "psychological wellbeing" in all fields of the database, combined with one of the following terms in the title or abstract: intervention or therapy or treatment or random* or control* or trial or RCT. Search strings were adapted to the according database. No limitations were used.

#### Study selection

Two data extractors (LAW and PDW) assessed the eligibility independently in a standardized manner. The retrieved records from the database search were screened by title and abstract. First, the extractors screened the first ten publications in PsycINFO together and discussed the results, and then both screened the next 100 studies in PsycINFO independently. They performed an interrater reliability check where Cohen’s kappa was 0.71, which is considered ‘good’ [36]. A consensus procedure for disagreement between them was established and disagreements were resolved by consensus. The remainder of the records were screened by the two researchers independently. After the titles and abstracts were screened for possible inclusion, full articles were assessed for eligibility.

### Data collection

#### Data items

Information was extracted from each included study on (1) study sample; (2) outcome measure (Ryff’s PWBS or MHC-SF) with number of items; (3) type of intervention; (4) number of sessions and treatment duration in weeks; (5) control group; (6) total sample size; (7) mean age of the sample with standard deviation or range; and (8) quality assessment.

#### Data collection process

LAW extracted the data from the included studies with a data extraction sheet, PDW checked the extracted data. Disagreements were resolved by discussion. We contacted 14 authors through e-mail for additional data. Seven authors responded and provided the unpublished data. In one case, data was obtained via the author of an earlier meta-analysis where the study was included. One author had lost the data due to a hard drive failure. For the remaining five articles, the authors did not respond. All in all, six studies could not be included due to missing data.

### Quality assessment

Quality was assessed with eight criteria, partly based on the criteria of the Cochrane collaboration [37] tailored for the included studies. (1) Was the randomization adequately described? (2) Were drop-out and reasons for drop-out properly described? (3) In case of drop-out, was an intention-to-treat analysis performed? (4) Were the professionals who delivered the intervention adequately qualified? (5) Was a power analysis carried out or were a total of at least 128 participants included (i.e., could the trial detect a moderate change according to a power analysis with Cohen’s d = .50, alpha = .05, power (1-beta) = .80)? (6) Was the treatment integrity checked? (7) Were the outcome measures at baseline assessed and study groups comparable? In the case of differences between groups, were adjustments made to correct for baseline imbalance? (8) Were inclusion/exclusion criteria described?

Each criterion was scored with 0 or 1. As certain criteria were not applicable to some studies, the percentage of items scored 1 across all applicable criteria was calculated. We classified study quality as lower (<40% quality index), intermediate (41–75%) or higher (>75%). For details, see Table in S1 Table. We included quality as a moderator in the moderator analysis, as we hypothesized that the effect size may differ between studies depending on the quality of the studies.

### Data analysis

All analyses were completed with the program Comprehensive Meta-Analysis (CMA, version 2.2.064).

We used the random effects model and a 95% confidence interval with two-tailed tests.

#### Summary measures

We expected considerable heterogeneity due to diverse intervention types and populations. Therefore, the meta-analysis was performed using a random effects model. If possible, outcomes from an intention-to-treat analysis were used. Samples for completers only were used when intention-to-treat samples were not provided. The primary outcome statistic was the standardized difference in means. For each study, between-group effect sizes were computed, using Cohen’s d. When Ryff’s PWBS were used, the six dimensions were joined in one outcome measure. Standard deviations were reconstructed from p-values or t- statistics when necessary. Lipsey’s rules for interpretation were used: small effect sizes range from 0 to 0.32, medium effect sizes range from 0.33 to 0.55 and large effect sizes are 0.56 or higher [38].

#### Heterogeneity

To evaluate between-study variability, we tested for heterogeneity with the chi-squared test Cochran’s Q and I^2^ statistics, which quantifies the amount of variation in results across studies, beyond the expected chance. The heterogeneity analysis was performed with a random effect model, a 95% confidence interval and a two-tailed test.

#### Moderators

Moderator analyses were conducted with the following moderators and categories: (1) *target group*: clinical (psychopathological or health problems) or non-clinical; (2) *age of target group*: adolescence/young adulthood (≤ 25 years), adulthood (26–55 years) or later life (≥55); (3) *intervention type*: self-help, individual face-to-face, or group face-to-face; (2) *number of sessions*: less (≤ 8 studies) or more (> 8 sessions); (5) *instrument*: PWBS or MHC-SF; (6) *control group*: not active (no treatment, waiting list, or care-as-usual) or active (placebo or alternative treatment); *(7) quality*: lower (<40%), intermediate (41% -75%) or higher quality (≥75%).

#### Publication bias

The risk of publication bias was estimated using a funnel plot, the Egger’s test and a trim and fill analysis.

#### Follow-up assessment

When available, between-group effect sizes (Cohen’s d) were computed for follow-up differences in psychological well-being.

## Results

### Study selection

**Fig 1:**
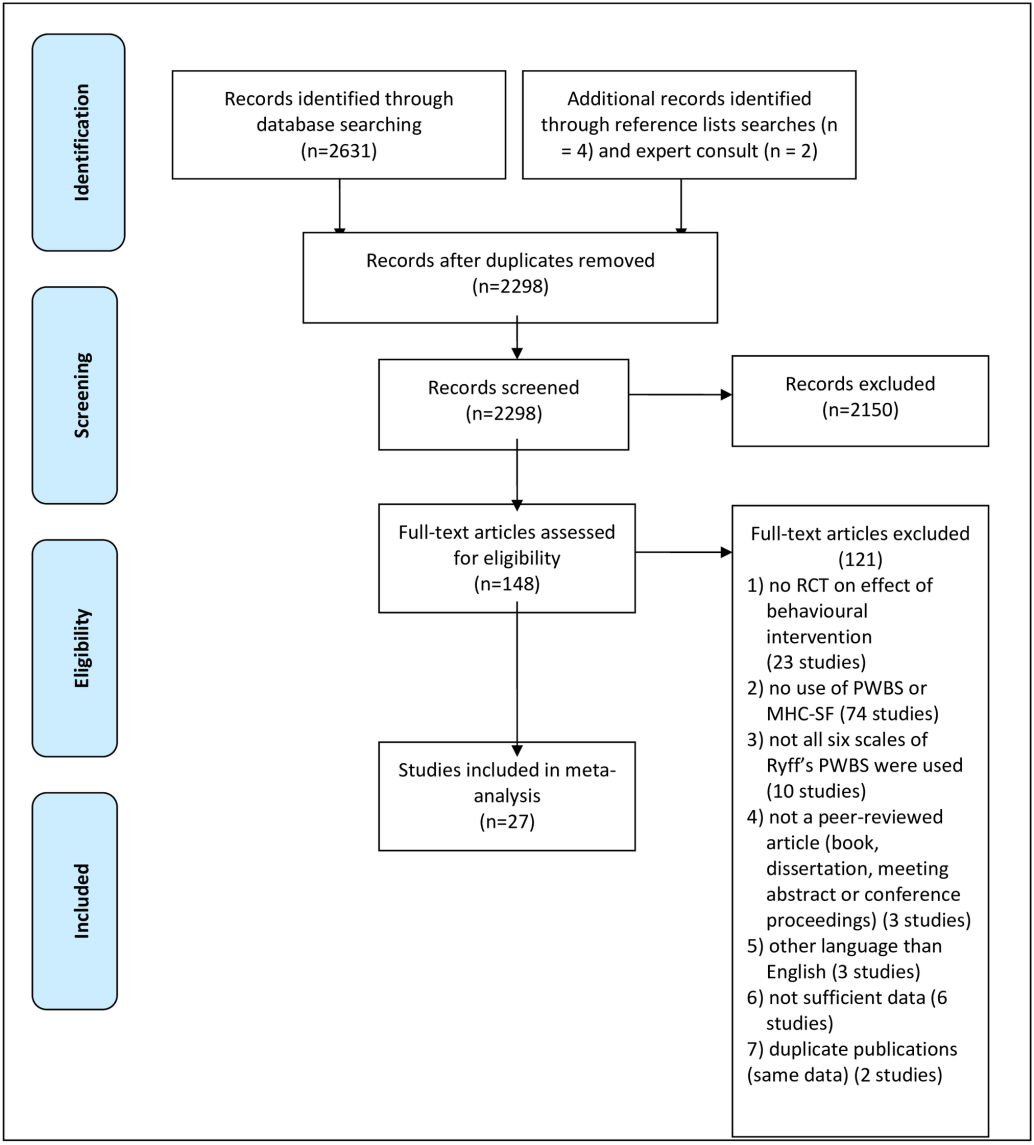
Flow diagram of the search and selection procedure of studies.

Fig 1 summarizes the database hits, (reasons for) exclusion and final inclusion in a flow diagram. We found 2631 records from Web of Science (1151), the Cochrane Library (1026), and PsycINFO (454), and Reference lists searches added four studies and expert consultation two studies. After adjusting for duplicates, 2298 studies remained and were screened for title and abstract. Of these, 2150 were discarded as the studies did not meet the inclusion criteria. The full texts of the remaining 148 studies were assessed for eligibility. 121 studies did not meet the inclusion criteria. Finally, a total of 27 studies met the inclusion criteria and were included in the meta-analysis.

### Study characteristics

The main characteristics of the studies are presented in Table 1. All 27 studies were RCTs published in peer-reviewed English journals. The studies were published between 1998 and 2014. The included studies involved 3579 participants. Sample size varied between 20 and 376 participants. Whereas 14 studies were conducted among non-clinical populations (e.g. employees, students), 13 studies used a clinical sample. The vast majority of the clinical samples had psychological disorders, mostly affective disorders. Only two studies used a population with physical complaints (i.e., hearing impairment [39] and chronic pain [40]). The mean age varied between 11 and 79 years. While 4 studies used adolescents or young adults, 18 studies examined adults, and 5 studies had a sample of older people. Interventions included well-being therapy, life review, positive psychology interventions, acceptance and commitment therapy, mindfulness interventions and identity interventions. Seven interventions were self-help (web-based or book), 6 were individually administered and 14 group-based. The duration of the interventions varied between 4 and 52 weeks. Whereas 15 studies had between four [41] and eight sessions, 10 studies had between 8 and 48 sessions [42]. Sixteen studies used the PWBS as outcome measure, 11 studies the MHC-SF. Six different versions of the PWBS were used, varying between 14 and 84 items. The control conditions included 16 non-active control groups (no intervention, waiting list, care-as-usual) and 13 active control groups (placebos such as relaxation sessions or alternative established interventions such as cognitive behavioral therapy). Nine studies were qualified as having a lower quality, 8 as intermediate and 10 as higher quality studies. Whereas 16 studies declared no conflict of interest [21, 22, 24, 39, 41–51], the other 11 studies did not mention whether there was a conflict of interest.

**Table 1:** Main characteristics of studies included in the meta-analysis.

1^st^ Author, year	Sample	Outcome measure, number of items	Intervention	Number of sessions, treatment duration in weeks	Control group	Total sample size	Mean age (SD or range)	Study quality
Addley, 2014 [43]	employees	MHC-SF, 14	1) assessment, health and wellbeing session, health coaching and web-bases lifestyle tools with online personal trainer, 2) assessment	(1) 9, 52, 2) 5, 52	no intervention	180	n.m.	intermediate
Afonso, 2011 [52]	elderly people with depressive symptomatology	PWBS, 84	individually administered reminiscence program	5, 5	1) no intervention, 2) placebo relaxation sessions	90	76 (6.7)	lower
Bolier, 2013 [44]	adults with depressive symptomatology	MHC-SF, 14	web-based self-help positive psychology intervention	24, 8	waiting-list	284	43.2 (11.8)	higher
Bolier, 2014 [45]	nurses and allied health professionals	MHC-SF, 14	web-based screening, tailored feedback and self-help interventions	4–8, 4–12	waiting-list	366	40 (11.9)	higher
Bonthuys, 2011 [53]	adults of a rural community in South Africa	MHC-SF, 14	holistic promotion of health in context	n.m.	n.m.	99	43 (20–83)	lower
Borness, 2013 [42]	employees	PWBS, 54	web-based cognitive training	48, 16	active control	135	41.3 (13.1)	higher
Fava, 1998 [46]	patients with affective disorders with residual symptoms	PWBS, 84	individually administered WBT	8, 16	CBT	20	28.3 (6.6)	lower
Fava, 2005 [24]	outpatients with generalized anxiety disorder	PWBS, 84	CBT and WBT group intervention	8, 16	CBT	20	41.9 (11.9)	lower
Fledderus, 2010 [21]	adults with psychological distress	MHC-SF, 14	ACT and mindfulness group intervention	8, 8	waiting-list	93	49 (24–71)	higher
Fledderus, 2012 [47]	adults with depressive symptomatology	MHC-SF, 14	ACT book self-help intervention with 1) minimal e-mail support, 2) extensive e-mail support	9, 9	waiting-list	376	42.5 (11.2)	higher
Green, 2006 [54]	normal, non-clinical population	PWBS, 14	life coaching group intervention	10, 10	waiting-list	56	42.7 (18–60)	lower
Goldstein, 2007 [55]	normal, non-clinical population	PWBS, 84	self-help exercise cultivating sacred moments	15, 3	writing task	83	n.m. (18–54)	lower
Hickson, 2007 [39]	older people with hearing impairments	PWBS, 24	active communication education group intervention	5, 5	placebo social program	178	73.9 (8.3)	intermediate
Josefsson, 2014 [56]	employees	PWBS, 18	mindfulness-based group intervention	7, 4	relaxation training	86	49.6 (10.3)	intermediate
Korte, 2012 [22]	older adults with depressive symptomatology	MHC-SF, 14	web-based guided self-help life review therapy	8, 12	waiting-list	202	63.3 (6.5)	higher
Lamers, 2014 [57]	middle-aged and older adults with depressive symptomatology	MHC-SF, 14	web-based guided self-help life review therapy	7, 10	1) expressive writing 2) waiting list	116	57 (9.5)	higher
Lee, 2010 [58]	middle-aged women with emotional distress	PWBS, 18	mindfulness and self-compassion group intervention	8, 8	waiting-list	75	40.9 (3.9)	lower
Meca, 2014 [59]	emerging adults, undergraduate psychology students	MHC-SF, 14	identity group intervention	5, 5	active group intervention	141	23.1 (2.2)	intermediate
Meléndez-Moral, 2013 [60]	elderly adults living in retirement homes	PWBS, n.m.	reminiscence group sessions	8, n.m.	no intervention	34	79.8 (8.7)	lower
Page, 2013 [61]	employees	PWBS, 42	positive psychology group program	6, 6	no intervention	23	37.7 (10)	intermediate
Pots, 2014 [48]	adults with depressive symptomatology	MHC-SF, 14	mindfulness-based cognitive group therapy	11, 11	waiting list	151	48 (11.3)	higher
Ruini, 2006 [41]	students	PWBS, 18	WBT school group intervention	4, 8	CBT	111	13.1 (0.7)	intermediate
Ruini, 2009 [49]	students	PWBS, 18	WBT school group intervention	6, 6	attention-placebo	227	14.4 (0.7)	intermediate
Spence, 2007 [62]	normal, non-clinical population	PWBS, 54	individual life coaching	10, 10	1) group peer coaching, 2) waiting list	41	39.3 (10.3)	lower
Stein, 2013 [50]	women with anorexia or bulimia	PWBS, 84	identity group intervention	40, 20	supportive psychotherapy	69	24 (4.1)	higher
Tomba, 2010 [51]	students from middle school	PWBS, 18	WBT group intervention	6, 6	anxiety management	162	11.41 (0.6)	intermediate
Trompetter, 2014 [40]	chronic pain sufferers	MHC-SF, 14	ACT web-based guided self-help intervention	9, 9–12	1) expressive writing, 2) waiting list	161	52.8 (12.6)	higher

ACT: acceptance and commitment therapy; CBT: cognitive behavioral therapy; CC: control condition; EC—experimental condition; MHC-SF: Mental Health Continuum—Short Form; n.a.: not applicable; n.m.: not mentioned; PWBS: Psychological Well-Being Scales; WBT: well-being therapy

### Results data analysis

#### Post-test effects

**Fig 2:**
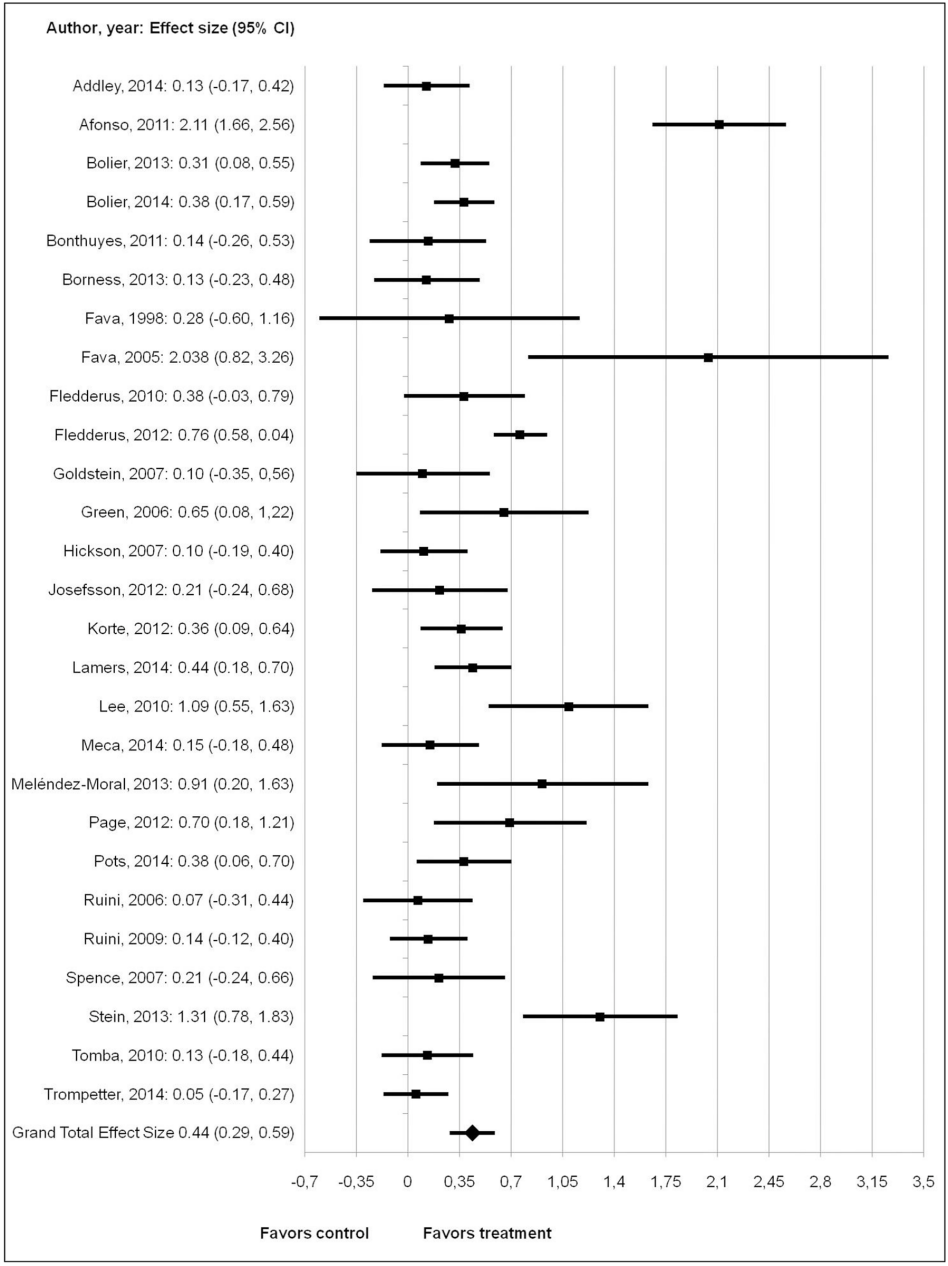
Forest plot for post-test effects of behavioral interventions on psychological well-being.

The random effect model showed that the behavioral interventions had a moderate effect on psychological well-being (Cohen’s d = 0.44; z = 5.62; p < .001). The 95% confidence interval was between 0.29 and 0.59, with a standard error of 0.08. The forest plot in Fig 2 displays the post-test effects.

#### Heterogeneity

Effect sizes of studies ranged from 0.05 to 2.11. A heterogeneity analysis revealed significant heterogeneity (Q (26) = 134.12; p < .001). Heterogeneity was high (I^2^ = 80.62). Therefore, moderator analyses were performed.

#### Moderators

Table 2 presents the findings of the moderator analyses. A systematic finding is that for 15 out of 17 categories, significant effects were found. No significant effects were found for age of target group, number of sessions, measurement instrument and control group. However, the strength of the effects differed for target group, intervention type and study quality. Interventions in clinical groups showed larger effects than those in non-clinical groups. Individual face-to-face interventions had stronger effects than self-help or group interventions. Studies of lower quality had higher effect sizes than studies of intermediate or higher quality. In a post-hoc analysis, we assessed whether the three significant moderating variables were interrelated among each other. There was no relation between target group and intervention type (*χ*^2^ = 1.1; df = 1; p = 0.587). Target group and study quality were related (*χ*^2^ = 9.4; df = 2; p = 0.009). Studies with clinical target groups had higher quality. The higher effects for clinical groups can thus not be attributed to a lower quality of studies. There was a significant relation between intervention type and quality of the study (*χ*^2^ = 14.1; df = 4; p = 0.007). Individual face-to-face interventions were more often assessed in studies with lower quality. Due to this contamination, it remains uncertain whether the intervention type or the quality of the study caused the higher effect sizes.

**Table 2:** Results of moderator analysis.

Variable	Value	Number of Studies	Std diff in means (95% CI)	Z-value(p-value)	Q-value (df),p-value
Target group	*Clinical*	13	0.63 (0.42, 0.84)	5.79 (<0.01)	
	*Non-clinical*	14	0.26 (0.06, 0.46)	2.49 (0.01)	
	*Total between*	27			6.11 (1), 0.013*
Age of target group	*Adolescence/ young adulthood*	4	0.12 (-0.25, 0.50)	0.65 (0.51)	
	*Adulthood*	18	0.44 (0.25, 0.63)	4.52 (<0.01)	
	*Later life*	5	0.72 (0.37, 1.06)	4.03 (<0.01)	
	*Total between*	27			5.18 (2), 0.075
Intervention type	*Self-help*	7	0.33 (0.05, 0.60)	2.34 (0.019)	
	*Individual*	6	0.90 (0.54, 1.26)	4.88 (<0.01)	
	*Group*	14	0.35 (0.14, 0.56)	3.26 (0.001)	
	*Total between*	27			7.64 (2), 0.022*
Number of sessions	*Less*	16	0.49 (0.28, 0.70)	4.51 (<0.01)	
	*More*	11	0.38 (0.14, 0.62)	3.14 (0.002)	
	*Total between*	27			0.41 (1), 0.53
Instrument	*PWBS*	16	0.54 (0.33, 0.76)	4.97 (<0.01)	
	*MHC-SF*	11	0.32 (0.1, 0.55)	2.79 (0.005)	
	*Total between*	27			1.92 (1), 0.166
Control group	*Not Active*	16	0.51 (0.31, 0.70)	5.08 (<0.01)	
	*Active*	14	0.40 (0.18, 0.62)	3.52 (<0.01)	
	*Total between*	30			0.54 (1), 0.461
Quality	*Lower*	9	0.75 (0.46, 1.03)	5.15 (<0.01)	
	*Intermediate*	8	0.19 (-0.07, 0.44)	1.46 (0.145)	
	*Higher*	10	0.43 (0.21, 0.66)	3.86 (<0.01)	
	*Total between*	27			8.36 (2), 0.015*

* significant (p < .05)

#### Publication bias

There is no clear indication of publication bias. Visual inspection of the funnel plot suggested no evidence of publication bias, as the distribution is symmetrical. Egger’s regression intercept also suggests that there is no publication bias (intercept = 1.53; t = 1.31; df = 25; p = 0.20). Duval and Tweedie’s trim and fill analysis indicated that no studies needed to be filled or trimmed, which suggests that the effect size was not affected by publication bias.

#### Follow-up effects

Twelve studies [21, 22, 40, 42, 44, 45, 49–51, 55, 57, 61] examined follow-up effects after at least 2 months up to 10 months. Nine of these 12 studies examined the follow-up at 6 months. The random effect model showed small but significant effects for psychological well-being, compared with a control group (Cohen’s d = 0.22; z = 4,9; p<0.001). The 95% confidence interval was between 0.13 and 0.31, with a standard error of 0.045. Heterogeneity was low (Q (11) = 11.45; p<0.41; I^2^ = 3.89).

## Discussion and Conclusion

Psychological well-being is increasingly used as an outcome in studies on behavioral interventions, besides measures of psychological complaints and psychopathological symptoms. Several studies reported evidence that psychological well-being can indeed be promoted through behavioral interventions. This is the first meta-analysis to assess their overall effect. A moderate effect size of 0.44 was found across studies for psychological well-being, with no indication for publication bias. Significant effects were found across the categories of the moderator variables, illustrating the systematic nature of the effects. In the follow-up assessment, the effect size was still significant, but small (0.22). This result has to be interpreted with caution as only 12 studies could be included in this analysis. It is important that future studies make use of follow-up measures to gain more insight in the longitudinal development of the effects of interventions on psychological well-being.

This study explicitly focused on psychological well-being as an integrated construct that builds on several psychological theories of the twentieth century. The effect size of psychological well-being is somewhat lower than the standardized mean difference of .61 that was reported in a meta-analysis by Sin and Lyubomirsky (11) and somewhat higher than the effect of .20 for psychological well-being in a meta-analysis by Bolier et al. [12]. These differences may be related to the fact that the first meta-analyses focused on subjective well-being whereas the second one included 10 different measures of psychological well-being in addition to the PWBS and MHC-SF, for example hope, mastery and purpose in life. This might demonstrate the importance of good definitions of well-being as different results may be obtained with instruments derived from different traditions. Furthermore, both previous meta-analyses focused on specific positive psychological interventions, whereas our study included a number of different therapeutic interventions. Because the interventions varied considerably, a reliable subgroup analysis was not possible. When sufficient studies will be published in the future, later meta-analyses could address differences between interventions, for example comparing positive psychological interventions, well-being therapy, acceptance and commitment therapy, and life review therapy. Despite the relatively high levels of stability of psychological well-being across time [19], these results show that it is possible to improve psychological well-being. Consequently, it might have more state-like characteristics, as a trait would be very hard to change, especially in a short period of time.

The heterogeneity was large with effects ranging from 0.05 [41] to 2.11 [52]. Although the statistical power is sufficient for the study in total, it is low for the moderator analyses [63]. Therefore, it is even more remarkable that we did find three significant moderators out of seven possible moderators. Effects were larger for clinical groups and in individual interventions. Interestingly, these moderators were also found significant in the meta-analyses of Sin and Lyubomirsky [11] and Bolier et al. [12]. The promotion of psychological well-being seem to be best suited for individuals who suffer from psychological or somatic complaints. One possible explanation is that clinical populations have more impaired levels of psychological well-being at the beginning of the intervention, indicating that there is more room for improvement. This finding is relevant because psychological well-being can be seen as an important component of recovery [64]. Higher levels of psychological well-being are associated with better physical health [15] and buffer against future disorders [16, 65], suggesting that people with higher levels are potentially more resilient [66, 67]. Furthermore, a personal approach with face-to-face contact appears to work better compared to self-help and group interventions. Yet interventions targeted at the general population or using self-help or group interventions showed smaller, but still significant effects. When such interventions have a large enough reach, they might also bring substantive public health gains [12].

For an interpretation of the results, it is important to be aware of possible limitations of the meta-analysis. First, one third of the studies had lower quality, whereas these studies also showed larger effects. However, the quality might have been underestimated, as it was scored conservatively: not reporting on the randomization procedures for example was rated as absence. Lower quality might also be attributed to the fact that new interventions were tested with pilot studies with a small number of participants. The larger effects of studies with lower quality might also contaminated with the finding that individual face-to-face interventions had higher effects. Future research needs RCTs with better quality, such as a larger number of participants based on a priori power analyses and longer follow-ups. Second, there are some limitations due to the search strategy. There was not sufficient data for six studies which met the inclusion criteria, limiting the completeness of the meta-analysis. The search strategy also may have been imperfect, as additional information sources revealed another six studies which were not found with the database search. Still, this possible limitation has been compensated by asking experts in the field and searching through reference lists of relevant articles and meta-analyses. We also excluded grey literature articles that were not peer-reviewed, which might have led to biased results. However, we did not find any indication of a publication bias. Another limitation is that the meta-analysis included highly heterogeneous studies; different outcomes may be due to factors such as different patient populations, protocol characteristics, and enrollment procedures [68, 69].

A broader point of discussion concerns the fact that the scales rely on self-reports. Self-reported well-being measures correlate with social desirability [70]. It would therefore be interesting to find new ways of measurements to assess aspects of psychological functioning in a more objective way, for example using biological markers or automatic behavioral analyses. Until the reliability and validity of such methods have been proven, the possible self-reporting biases should be kept in mind when interpreting results of meta-analyses such as the current one.

Despite the limitations, we conclude that psychological well-being can be significantly improved to a moderate extent. This is important evidence for the development and implementation of interventions and policies in the field of mental health promotion. Improvement of psychological well-being is especially successful in clinical populations. Based on this meta-analysis, individual face-to-face interventions can be considered as valuable option when developing interventions for an improved psychological well-being. There is a need for higher quality studies in this emerging field to be able to further underpin the promising results of this meta-analysis.

## Supporting Information

S1 Checklist (DOC)

S1 Table Methodological Quality Assessment Criteria. (PDF)
