# Supplementary material for: Can We Increase Psychological Well-Being? The Effects of Interventions on Psychological Well-Being: A Meta-Analysis of Randomized Controlled Trials
Source: PLoS One. 2016 Jun 21;11(6):e0158092. doi: 10.1371/journal.pone.0158092 (PMC4915721; doi:10.1371/journal.pone.0158092)
Supplement: S1 Table — (PDF) [file pone.0158092.s002.pdf]

S 1. Tabel. Methodological Quality Assessment Criteria.

Criteria	1	0
Randomization	Adequate description of randomization in text about how the randomization was done.	Randomization not described or only ‘participants were randomly assigned’.
Description of drop-out	Drop-out was described (numbers and reason of drop-out) or dropout analysis was performed, or a flow chart has been included, or there was no dropout.	No description of drop-out, or only stating that there was drop-out without giving numbers, or not stating the reasons.
Intention to treat analysis	Intention to treat analysis was performed, or there were no dropouts.	Completers-only analysis was used or it was not stated.
Qualified professionals	At least one of the professionals was experienced or trained or was a schooled psychologist / psychiatrist or health professional.	Specific experience or training was not reported.
Power analysis / $n \geq 128$	Adequate power analysis or a total of minimal 128 participants (moderate effect of 0.5, t-test, 2-sided, alpha 0.05, power 0.8).	No power analysis and less than 128 participants in total or calculated power was not reached.
Treatment integrity	Treatment integrity was checked (supervision of the professionals during the intervention or recording of the sessions or systematic screening of protocol adherence).	Treatment integrity not checked or not reported.
Baseline comparability	Comparability of the outcome measures at baseline. It was explicitly assessed if study groups were comparable at the beginning of the study. It was stated if the groups are comparable or not. In the case of differences between groups at baseline, adjustments were made to correct for baseline imbalance by using appropriate covariates.	Imbalance that was not adjusted for or baseline comparability was not assessed.
Inclusion/exclusion	Inclusion or exclusion criteria were adequately described.	No description for inclusion or exclusion criteria.
